# Cortical Morphological Networks Differ Between Gyri and Sulci

**DOI:** 10.1007/s12264-024-01262-7

**Published:** 2024-07-23

**Authors:** Qingchun Lin, Suhui Jin, Guole Yin, Junle Li, Umer Asgher, Shijun Qiu, Jinhui Wang

**Affiliations:** 1 Institute for Brain Research and Rehabilitation, South China Normal University, Guangzhou, 510631 China; 2 Department of Air Transport, Faculty of Transportation Sciences, Czech Technical University in Prague (CTU), Prague, 128 00 Czech Republic; 3 School of Interdisciplinary Engineering and Sciences (SINES), National University of Science and Technology (NUST), Islamabad, 44000 Pakistan; 4 Department of Radiology, The First Affiliated Hospital of Guangzhou University of Chinese Medicine, Guangzhou, 510405 China; 5 Key Laboratory of Brain, Cognition and Education Sciences, Ministry of Education, Guangzhou, 510631 China; 6 Center for Studies of Psychological Application, South China Normal University, Guangzhou, 510631 China; 7 Guangdong Key Laboratory of Mental Health and Cognitive Science, South China Normal University, Guangzhou, 510631 China

**Keywords:** Morphological connectivity, Magnetic resonance imaging, Test-retest reliability, Cortical folding, Graph theory

## Abstract

This study explored how the human cortical folding pattern composed of convex gyri and concave sulci affected single-subject morphological brain networks, which are becoming an important method for studying the human brain connectome. We found that gyri-gyri networks exhibited higher morphological similarity, lower small-world parameters, and lower long-term test-retest reliability than sulci-sulci networks for cortical thickness- and gyrification index-based networks, while opposite patterns were observed for fractal dimension-based networks. Further behavioral association analysis revealed that gyri-gyri networks and connections between gyral and sulcal regions significantly explained inter-individual variance in Cognition and Motor domains for fractal dimension- and sulcal depth-based networks. Finally, the clinical application showed that only sulci-sulci networks exhibited morphological similarity reductions in major depressive disorder for cortical thickness-, fractal dimension-, and gyrification index-based networks. Taken together, these findings provide novel insights into the constraint of the cortical folding pattern to the network organization of the human brain.

**Supplementary Information:**

The online version contains supplementary material available at 10.1007/s12264-024-01262-7.

## Introduction

The human brain works as an interconnected complex network, which can be mapped through multimodal magnetic resonance imaging (MRI) technologies *in vivo* [1]. By analyzing the network with graph-based approaches, previous studies have found several nontrivial topological properties, such as small-world organization, modular structure, and highly connected hubs [2–4]. These findings provide crucial insights into the organizational principles that govern the wiring diagram in the human brain.

However, the nontrivial topological properties of the human brain network are not uniformly distributed over the cortical mantle. For example, significant differences have been observed in the small-world organization between the left and right brain hemispheres [5–7]. Beyond the brain hemisphere, there is another important factor that is increasingly found to affect the human brain network topology, namely, the highly convoluted cortical folding pattern. The cortical folding pattern is composed of convex gyri and concave sulci, which is one of the most prominent features of human brain morphology. Evidence from structural and functional MRI studies has indicated that the human brain network composed of gyral regions has stronger connectivity and higher network efficiency than that composed of sulcal regions [8–14]. Despite these progresses, little is known regarding the gyral and sulcal effects on the human brain network derived from structural MRI data (i.e., morphological brain network).

The morphological brain network depicts the pattern of inter-regional similarity in local brain morphology. Early studies estimate inter-regional morphological similarity by calculating the Pearson correlation coefficient in a certain morphological feature across a cohort of participants [15, 16]. The population-based method generates only one network for a participant group, constraining their broader applicability. With advances in methodology, a morphological brain network can now be built at the individual level based on a single structural MRI image [17]. Single-subject morphological brain network has proven as a reliable and biologically meaningful means to study the human brain connectome [18].

In this study, we aimed to provide a comprehensive analysis of the gyral and sulcal effects on the single-subject morphological brain network. Specifically, we adopted our previous method [19] to construct four types of single-subject morphological brain networks for multiple independent datasets. These datasets were used to examine the differences between gyral and sulcal morphological brain networks from different aspects, including inter-regional morphological similarity, small-world organization, test-retest (TRT) reliability, ability to account for inter-individual variance in behavior and cognition, and susceptibility to major depressive disorder (MDD). Based on previous studies, we hypothesize that gyral and sulcal morphological brain networks would differ in all aspects examined above.

## Materials and Methods

### Participants and Data Collection

This study included four independent datasets: the Human Connectome Project (HCP) dataset [20], the Beijing Normal University (BNU) TRT dataset [21], the Southwestern University (SWU) TRT dataset [22], and an MDD dataset [23, 24]. The HCP dataset was used to study the differences in inter-regional morphological similarity, small-world organization, and ability to explain inter-individual variance in behavior and cognition between gyral and sulcal morphological brain networks; the BNU dataset was used to examine the differences in the short-term TRT reliability between gyral and sulcal morphological brain networks; the SWU dataset was used to examine the differences in the long-term TRT reliability between gyral and sulcal morphological brain networks; and the MDD dataset was used to explore whether gyral and sulcal morphological brain networks were differentially altered in MDD.

#### HCP Dataset

The HCP dataset contained a total of 1,113 participants, for whom T1-weighted structural MRI scans were acquired. The structural images were scanned on a Siemens Skyra Connectome scanner with a magnetization-prepared rapid-gradient echo sequence: repetition time (TR) = 2,400 ms; inversion time (TI) = 1,000 ms; echo time (TE) = 2.14 ms; voxel size = 0.7 mm × 0.7 mm × 0.7 mm; field of view (FOV) = 224 mm × 224 mm; flip angle (FA) = 8°; and bandwidth (BW) = 210 Hz/pixel. Out of the 1,113 participants, 444 were included in this study, who were unrelated with respect to their family structure (male/female: 205/239; age range: 22–35 years old). In addition, the HCP dataset included a broad range of behavioral and cognitive measures that were evaluated mainly *via* the NIH Toolbox. Similar to our previous study [25], a total of 60 items were screened out from the original 581 items, which belonged to six domains, including Alertness, Cognition, Emotion, Motor, Personality, and Sensory (Table S1). The items within each domain were averaged after the standardization of each item to make the scale comparable (*Z*-score transformation across participants).

#### BNU Dataset

The BNU dataset included 57 healthy participants [male/female: 30/27; mean age: 23.05 ± 2.29 (SD) years old, range: 19–30 years old], all of whom completed two MRI scan sessions. The mean interval between the two sessions was 40.94 ± 4.51 (SD) days (range: 33–55 days). There were no behavioral data available for this dataset. All participants were scanned on a 3T Siemens Trio-Tim MRI scanner (Siemens Healthcare, Erlangen, Germany) with a fast gradient echo sequence: TR = 2,530 ms; TI = 1,100 ms; TE = 3.39 ms; slice thickness = 1.33 mm; number of slices = 144; no inter-slice gap; FOV = 256 mm × 256 mm; and FA = 7°.

#### SWU Dataset

The SWU dataset included 121 healthy participants (male/female: 60/61; mean age at time point 1: 19.68 ± 0.95 (SD) years old, range: 17–22 years old), all of whom completed three MRI scan sessions. The mean interval was 313.39 ± 122.09 (SD) days (range: 120–653 days) between session 1 and session 2, 556.92 ± 67.64 (SD) days (range: 439–699 days) between session 2 and session 3, and 870.31 ± 116.58 (SD) days (range: 697–1,201 days) between session 1 and session 3. Although some behavioral data were available for this dataset, they were not used in this study. All participants were scanned on a 3T Siemens Trio-Tim MRI scanner (Siemens Medical, Erlangen, Germany) at the Imaging Center of SWU with a fast gradient echo sequence: TR = 1,900 ms; TI = 900 ms; TE = 2.52 ms; matrix = 256 × 256; slice thickness = 1 mm; number of slices = 176; voxel size = 1 mm × 1 mm × 1 mm; and FA = 9^°^.

#### MDD Dataset

The MDD dataset included 100 first-episode, drug-naive MDD patients (male/female: 34/66; mean age: 29.46 ± 9.34 (SD) years old, range: 18–55 years old) and 99 healthy controls (male/female: 41/58; mean age: 29.59 ± 10.33 (SD) years old, range: 20–55 years old). There were no significant differences in age, sex, or education between the two groups. For the patients, the mean Hamilton Depression Rating Scale (HAMD) score was 22.15 ± 3.18 (SD) (range: 18–36) and the mean course of the disease was 8.64 ± 10.86 (SD) months (range: 0.5–60 months). All participants were scanned on a GE Signa HDx 3.0T MRI scanner (GE Healthcare, Milwaukee, USA) with a 3-dimension Bravo sequence at The First Affiliated Hospital, Guangzhou University of Chinese Medicine, Guangdong, China. Please refer to our previous study [23] for details on the diagnosis of MDD and structural MRI imaging parameters. Notably, the imaging parameters differed slightly between participants. Thus, the ComBat harmonization approach [26] was utilized to control for the potential effects of different imaging parameters on morphological brain networks.

### Structural Image Preprocessing

In this study, we constructed single-subject morphological brain networks with our previously developed method, which was originally established for cortical thickness (CT), fractal dimension (FD), gyrification index (GI), and sulcus depth (SD) [19]. These four morphological features are widely used in literature to study brain development, aging, and disease [27–30], and show differential developmental and aging trajectories and alteration patterns in brain diseases [31–34]. In addition, these four morphological features can all be computed by the CAT12 toolbox (http://www.neuro.uni-jena.de/cat) in a fast way. Thus, these four morphological features were used in this study.

Specifically, similar to our previous studies [19, 33, 35, 36], a standard pipeline from the CAT12 Toolbox was applied to individual structural images to derive four surface-based, vertex-wise cortical morphological maps in subject native space (i.e., CT, FD, GI, and SD). Briefly, CT was estimated using a fast and reliable projection-based thickness method, and the others were estimated based on spherical harmonic reconstructions. These cortical morphological maps were further resampled into the common fsaverage template, and smoothed using a Gaussian kernel. According to the CAT12 manual, individual CT maps were smoothed using a Gaussian kernel with 12-mm full width at half maximum, while the other maps were smoothed using a Gaussian kernel with 25-mm full width at half maximum. The usage of larger filter sizes for the FD, GI, and SD maps is due to the underlying nature of these folding measures that reflect contributions from both sulci and gyri. Therefore, the filter size should exceed the distance between a gyral crown and a sulcal fundus. Notably, for the BNU and SWU datasets, the “Segment Longitudinal” module was used for tissue segmentation of individual structural images.

### Construction of Single-Subject Morphological Brain Networks

#### Brain Parcellation

We utilized the Destrieux atlas [37] to divide the cortical surface into 74 regions of interest (ROIs) in each hemisphere, which were composed of 29 gyral regions, 31 sulcal regions, and 14 ambiguous regions. We excluded the ambiguous regions, and a total of 60 regions were finally included in each hemisphere (Table S2).

#### Morphological Similarity Estimation

Similar to our previous studies [19, 33, 35, 36, 38], a Jensen-Shannon divergence (JSD)-based approach was used to estimate inter-regional morphological similarity. Briefly, a probability density function was first derived for each morphological feature of each ROI. The probability density functions were then converted to probability distribution functions (PDFs), and used to estimate the JSD between regions. Formally, for two PDFs *P* and *Q*, the JSD was calculated as:

$$\text{JSD}(P||Q) = \frac{1}{2}\text{KLD} (P||\frac{P+Q}{2})+\frac{1}{2}\text{KLD}(Q||\frac{P+Q}{2})$$

where $\text{KLD}(P||Q) = {\sum }_{i = 1}^{n}P(i)\text{log}\frac{P(i)}{Q(i)}$ with *n* being the number of sample points (2^8^ in this study) [39]. Finally, the JSD was transformed to a similarity measure calculated as the square root of the JSD, followed by subtracting it from 1. After calculating the JSD-based similarity between each pair of ROIs, we obtained 4 types of morphological brain networks (i.e., CT-based network, CTN; FD-based network, FDN; GI-based network, GIN; SD-based network, SDN) for each structural image that were composed of both gyral and sulcal regions (120 × 120; termed as GS-GS networks). Each of the GS-GS networks was further divided into three subcomponents: gyri-gyri (G-G) network (58 × 58), sulci-sulci (S-S) network (62 × 62), and gyri-sulci (G-S) network (58 × 62).

### Network Analysis

#### Threshold Selection

For the HCP dataset, a proportional (i.e., sparsity-based) thresholding approach was adopted to exclude edges with low morphological similarity in the G-G and S-S morphological brain networks. This thresholding approach improves the TRT reliability of morphological brain networks [35], and more importantly, ensures the comparability of the topological organization between networks with different sizes [40]. Sparsity is defined as the ratio of the number of actual edges divided by the maximum possible number of edges in a network. For an undirected, binary network as is the case for this study, the maximum possible number of edges is calculated as *N* × (*N*−1)/2, where *N* is the number of nodes in the network. Owing to the lack of a definitive way to select a single sparsity, the G-G and S-S morphological brain networks of each participant were repeatedly thresholded over a consecutive sparsity range of [0.08 0.4] with an interval of 0.02. The lower limit was determined to ensure that the resultant binary networks were estimable for the small-world attributes [41]. That is, for each binary network, the average degree over all nodes should be larger than 2 × log(*N*), with *N* being 58 for the gyral networks and 62 for the sulcal networks. According to this criterion, the lower limit of the sparsity range was 0.071 for the gyral networks and 0.068 for the sulcal networks. To exclude possible effects of this difference on the comparison of topological organization between the gyral and sulcal morphological brain networks, the lower limit of the sparsity range was finally set to 0.08. As for the upper limit of the sparsity range, it was empirically chosen to guarantee that the resultant binary networks had sparse properties [40, 42].

#### Small-World Parameters

The small-world parameters include clustering coefficient, *C*_*p*_, and characteristic path length, *L*_*p*_ [41]. *C*_*p*_ quantifies the local interconnectivity of a network, and *L*_*p*_ is an indicator of the overall routing efficiency of a network. We calculated the small-world parameters for each G-G and S-S morphological brain network with the GRETNA toolbox [43]. The small-world parameters were further normalized by corresponding parameters derived and averaged >100 matched random networks. These random networks were generated using a topological rewiring method [44] to guarantee the same degree distributions as the real brain networks. Typically, a small-world network should fulfill the following conditions: normalized *C*_*p*_ >1 and normalized *L*_*p*_ ~1. Notably, since the small-world parameters were calculated as a function of sparsity, we further computed the area under the curve (i.e., the integral over the entire sparsity range) for each parameter, which was used to simplify subsequent statistical analysis [45].

### TRT Reliability

For the BNU and SWU datasets, we utilized a common index of intra-class correlation (ICC) [46] to assess the TRT reliability of each edge in the G-G and S-S morphological brain networks. Specifically, for a given edge, the ICC was calculated as:

$$\text{ICC }= \frac{{\text{MS}}_{b }- {\text{MS}}_{w}}{{\text{MS}}_{b} + \left(k-1\right) {\text{MS}}_{w}}$$

where ${\text{MS}}_{b}$ is the between-subject sum of squares, ${\text{MS}}_{w}$ is the within-subject sum of squares, and $k$ is the number of repeated observations per participant. Notably, for the SWU dataset, the ICC was calculated separately between sessions 1 and 2, sessions 1 and 3, and sessions 2 and 3. Finally, we obtained 1 short-term and 3 long-term TRT reliability matrices for each type of G-G and S-S morphological brain networks. In accordance with our previous studies [19, 47], the TRT reliability values were classified into poor (0 < ICC <0.25), low (0.25 < ICC <0.4), fair (0.4 < ICC <0.6), good (0.6 < ICC <0.75), and excellent (0.75 < ICC <1.0).

### Behavioral and Cognitive Associations

For the HCP dataset, a multivariate variance component model [48, 49] was used to examine behavioral and cognitive associations of the GS-GS morphological brain networks. Assuming that behavioral and cognitive variance is the sum of the effect of the GS-GS morphological brain networks, ***B***, and environmental effect, ***E***, the multivariate model is:

$${\varvec{Y}} = {\varvec{B}}+{\varvec{E}}, \;{\varvec{B}} \sim N\left(0, {\sigma }_{\text{b}}^{2}{\varvec{M}}\right), \;{\varvec{E}} \sim N(0, {\sigma }_{\text{e}}^{2}{\varvec{I}})$$

where ${\varvec{Y}}$ is a $N\times P$ matrix including the behavioral and cognitive data of all participants, ${\sigma }_{\text{b}}^{2}$ and ${\sigma }_{\text{e}}^{2}$ represent the variance of the GS-GS morphological brain networks and environment, respectively, ${\varvec{M}}$ is a correlation matrix of the GS-GS morphological brain networks between participants, and ${\varvec{I}}$ is an identity matrix. The multivariate model follows distributional assumptions of $\text{vec}\left({\varvec{B}}\right) \sim {N}\left(0, {\sum }_{{\varvec{B}}}\otimes {\varvec{M}}\right)$ and $\text{vec}\left({\varvec{E}}\right) \sim {N}\left(0, {\sum }_{{\varvec{E}}}\otimes {\varvec{I}}\right)$, where $\text{vec}\left(.\right)$ is the matrix vectorization operator that converts a matrix into a vector, $\otimes$ denotes the Kronecker product of matrices, and ${\Sigma }_{{\varvec{B}}}$ and ${\Sigma }_{{\varvec{E}}}$ are $P \times P$ matrices estimated from ${\varvec{M}}$ and ${\varvec{Y}}$. The behavioral and cognitive variance explained by the GS-GS morphological brain networks is evaluated as:

$$V = \frac{\text{tr}({\Sigma }_{{\varvec{B}} })}{\text{tr}({\Sigma }_{{\varvec{P}} })} = \frac{\text{tr}({\Sigma }_{{\varvec{B}} })}{\text{tr}\left({\Sigma }_{{\varvec{B}}\boldsymbol{ }}\right)+\text{tr}({\Sigma }_{{\varvec{E}} })}$$

where ${\Sigma }_{{\varvec{P}}}$ is the behavioral and cognitive covariance matrix, $\text{tr}(.)$ is the trace operator of a matrix, and $V$ indicates how much inter-individual behavioral and cognitive variance is explained by inter-individual variance in the GS-GS morphological brain networks.

To examine whether the observed proportions of behavioral and cognitive variance explained by the GS-GS morphological brain networks could occur by chance, we randomly shuffled the real behavioral and cognitive data, and reran the multivariate variance component model (1,000 times). These procedures generated a set of null distributions, based on which a *P-*value was obtained for the association analysis between each behavioral and cognitive domain and the GS-GS morphological brain networks. A false discovery rate (FDR) procedure was used to correct for multiple comparisons across behavioral and cognitive domains at the level of *q* <0.05. If a significant association was observed, we further performed an edgewise correlation analysis to locate edges that were significantly correlated to the corresponding behavioral and cognitive domain. The threshold-free network-based statistics (TFNBS) algorithm [50] was used to correct for multiple comparisons across edges.

In addition, we ran the multivariate variance component model for each subcomponent of the GS-GS morphological brain networks.

### Statistical Analysis

#### Differences in the Morphological Similarity Between Gyral and Sulcal Networks

For the HCP dataset, we first examined the differences in the mean morphological similarity between the three subcomponents of the GS-GS morphological brain networks using nonparametric permutation tests. Then, for each gyral (or sulcal) region, we performed a nonparametric permutation test to examine the difference between its mean morphological similarity with the other gyral (or sulcal) regions and its mean morphological similarity with all sulcal (or gyral) regions. Multiple comparisons were corrected using the FDR procedure across gyral (or sulcal) regions at the level of *q* <0.05.

#### Differences in the Small-World Parameters Between Gyral and Sulcal Networks

For the HCP dataset, nonparametric permutation tests were used to examine the differences in the area under the curve for the small-world parameters (normalized *C*_*p*_ and normalized *L*_*p*_) between the G-G and S-S morphological brain networks. To examine whether the differences were dependent on the choice of sparsity thresholds, we further compared the small-world parameters between the G-G and S-S networks at each sparsity threshold using nonparametric permutation tests. Multiple comparisons were corrected using the FDR procedure across different sparsity thresholds at the level of *q* <0.05.

#### Differences in the TRT Reliability Between Gyral and Sulcal Networks

For the BNU and SWU datasets, nonparametric permutation tests were used to examine the differences in the TRT reliability (short-term and long-term) between the G-G and S-S morphological brain networks.

#### Differences in the Morphological Similarity Between the MDD Patients and Controls

For the MDD dataset, nonparametric permutation tests were used to examine the differences in the mean morphological similarity in the GS-GS networks and each of their subcomponents between the patients and controls. During between-group comparisons, the effects of age and sex were controlled *via a* general linear model. For measures showing significant between-group differences, we further examined their clinical relevance by calculating them with the HAMD scores and course of disease of the patients.

## Results

### Different Levels of Morphological Similarity Between Gyral and Sulcal Morphological Brain Networks

**Fig. 1:**
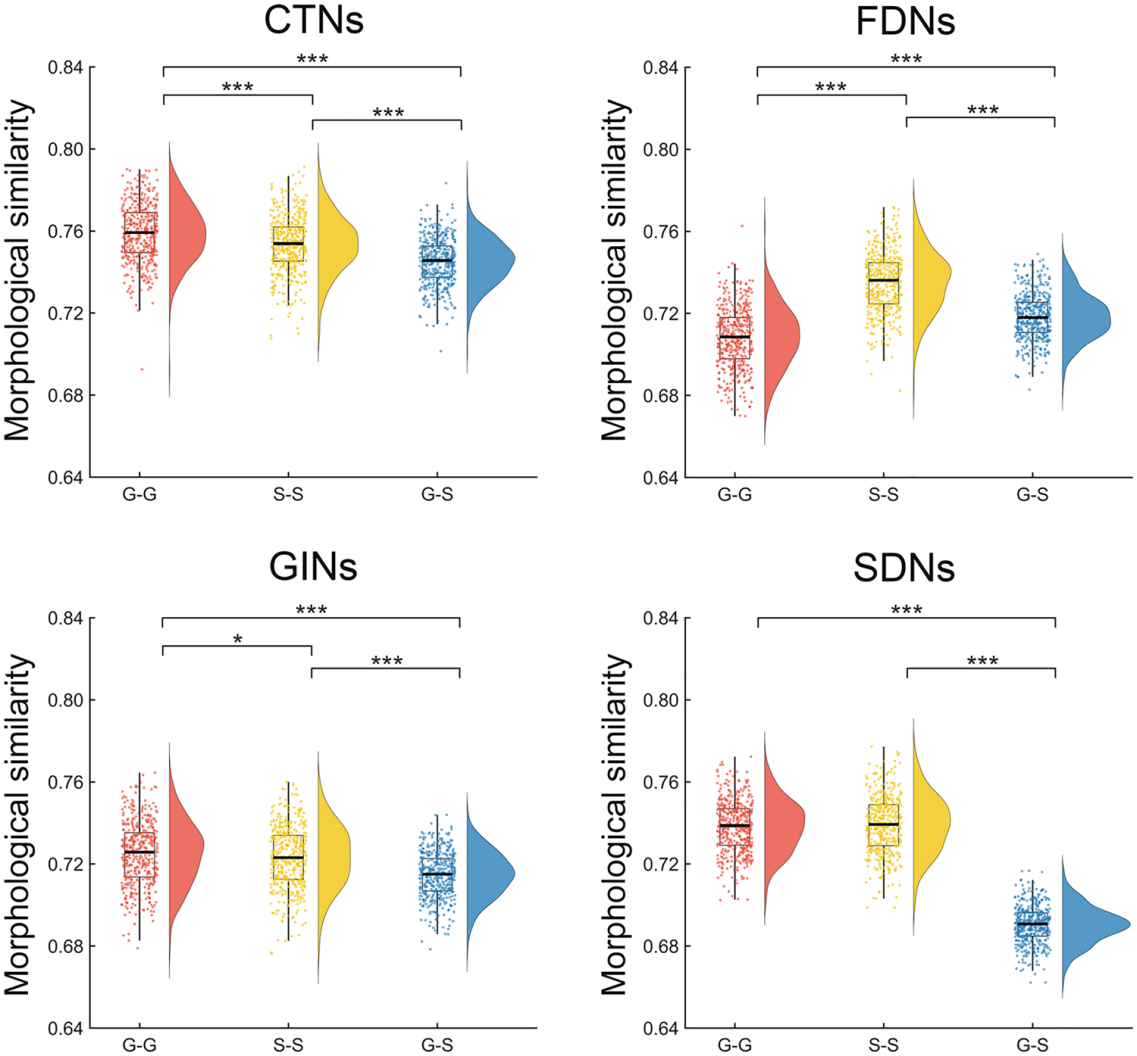
Differences in the mean morphological similarity between the three subcomponents of the GS-GS morphological brain networks. Significant differences were observed among the three subcomponents for each of the four types of morphological brain networks. CTNs, cortical thickness-based networks; FDNs, fractal dimension-based networks; GINs, gyrification index-based networks; SDNs, sulcal depth-based networks; G-G, gyri-gyri; S-S, sulci-sulci; G-S, gyri-sulci. *P < 0.05; ***P <0.001 (permutation test; 444 G-G networks *vs* 444 S-S networks *vs* 444 G-S networks).

Significant differences were observed in the mean morphological similarity between the three subcomponents of the GS-GS morphological brain networks (Fig. 1). Specifically, for the CTNs and GINs, the highest morphological similarity was observed for the G-G networks, followed by the S-S networks and G-S networks (all *P* <0.05); for the FDNs, the highest morphological similarity was observed for the S-S networks, followed by the G-S networks G-G networks (all *P* <0.001); for the SDNs, the morphological similarity was comparable between the G-G and S-S networks (*P* = 0.298), both of which were larger than the G-S networks (both *P* <0.001).

**Fig. 2:**
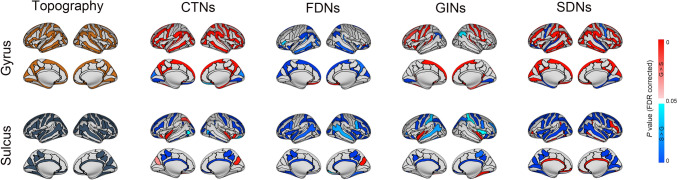
Regions showing significant differences between their mean morphological similarities with gyral and sulcal regions. For most gyral regions, they were found to show significantly higher morphological similarity with other gyral regions than sulcal regions. Similar results were observed for most sulcal regions. CTNs, cortical thickness-based networks; FDNs, fractal dimension-based networks; GINs, gyrification index-based networks; SDNs, sulcal depth-based networks; G, gyri; S, sulci.

At the nodal level, a total of 86 (49 gyri + 37 sulci), 85 (36 gyri + 49 sulci), 68 (29 gyri + 39 sulci), and 118 (58 gyri + 60 sulci) regions were found to show significant differences between their mean morphological similarity with gyral and sulcal regions for the CTNs, FDNs, GINs, and SDNs, respectively (*P* <0.05, FDR corrected) (Fig. 2). The differences were mainly characterized by higher intra-class (i.e., G-G or S-S) than inter-class (i.e., G-S) morphological similarity for the CTNs (71, 82.3%), GINs (57, 83.8%), and SDNs (92, 78.0%). For the FDNs, however, the higher intra-class than inter-class morphological similarity was observed mainly for sulcal regions (48, 98.0%); with regard to gyral regions, most were more highly connected to sulcal than gyral regions (31, 86.1%).

### Different Small-world Parameters Between Gyral and Sulcal Morphological Brain Networks

**Fig. 3:**
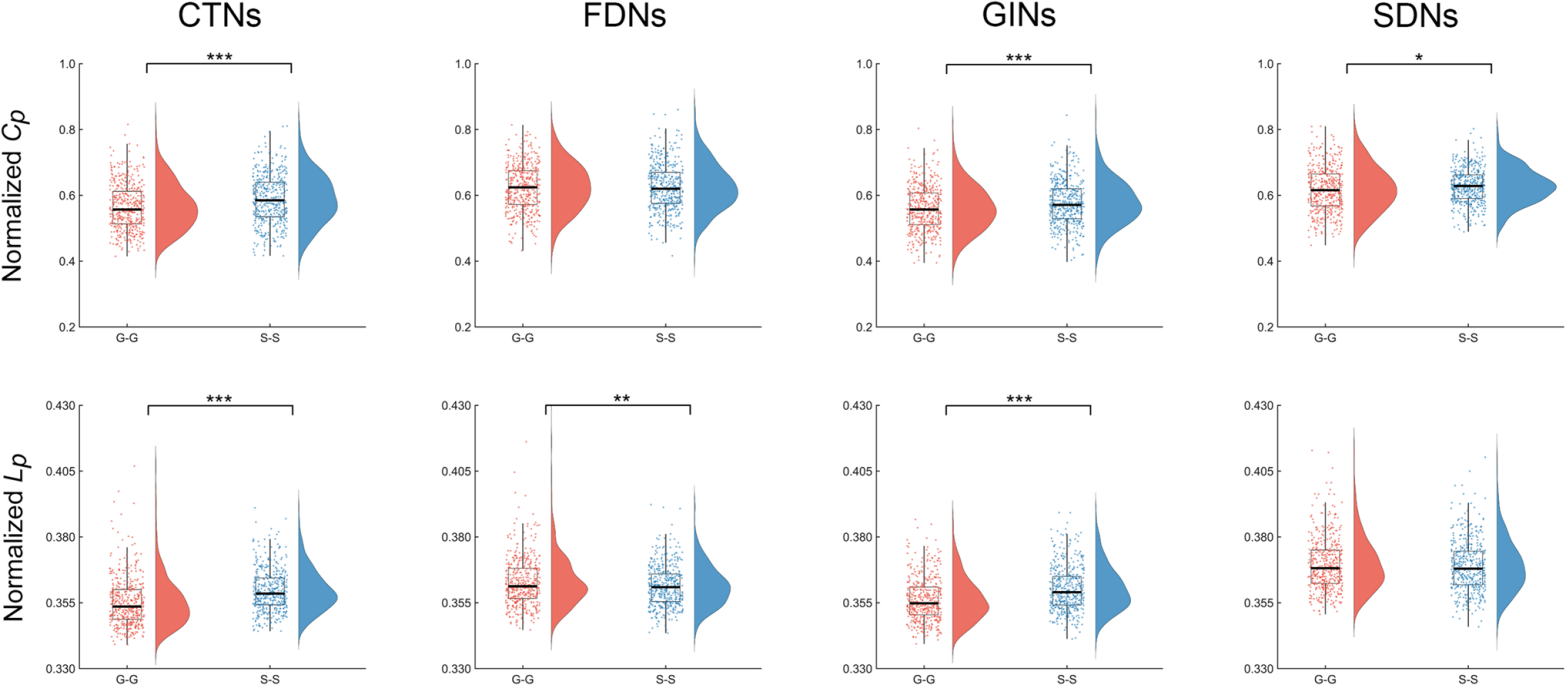
Differences in the small-world parameters between gyral and sulcal morphological brain networks. Compared to the sulcal networks, the gyral networks showed a significantly lower normalized clustering coefficient for the CTNs, GINs, and SDNs, significantly lower normalized characteristic path length for the CTNs and GINs, and significantly higher normalized characteristic path length for the FDNs. CTNs, cortical thickness-based networks; FDNs, fractal dimension-based networks; GINs, gyrification index-based networks; SDNs, sulcal depth-based networks; G-G, gyri-gyri; S-S, sulci-sulci; *C*_*p*_, clustering coefficient; *L*_*p*_, characteristic path length. *P < 0.05; **P < 0.01; ***P < 0.001 (permutation test; 444 G-G networks *vs* 444 S-S networks).

Both the G-G and S-S morphological brain networks exhibited typical small-world organization (i.e., normalized *C*_*p*_ >1 and normalized *L*_*p*_ ~1) over the entire sparsity range regardless of the morphological feature based on which the networks were constructed (Fig. S1). Nevertheless, compared to the G-G networks, the S-S networks showed significantly higher normalized *C*_*p*_ for the CTNs (*P* <0.001), GINs (*P* <0.001), and SDNs (*P* = 0.011), significantly higher normalized *L*_*p*_ for the CTNs (*P* <0.001) and GINs (*P* <0.001), and significantly lower normalized *L*_*p*_ for the FDNs (*P* = 0.009) (Fig. 3). Further comparisons at each sparsity threshold revealed that all differences derived based on the area under the curve were observed at almost all sparsity thresholds except for the lower normalized *L*_*p*_ in the S-S than G-G networks for the FDNs (Fig. S2). In addition, additional differences were observed in normalized *L*_*p*_ for the SDNs, characterized by significantly higher values at low sparsity thresholds (0.08–0.16) but lower values at high sparsity thresholds (0.3–0.4) for the G-G than S-S networks (Fig. S2).

### Varying Degrees of TRT Reliability Between Gyral and Sulcal Morphological Brain Networks

**Fig. 4:**
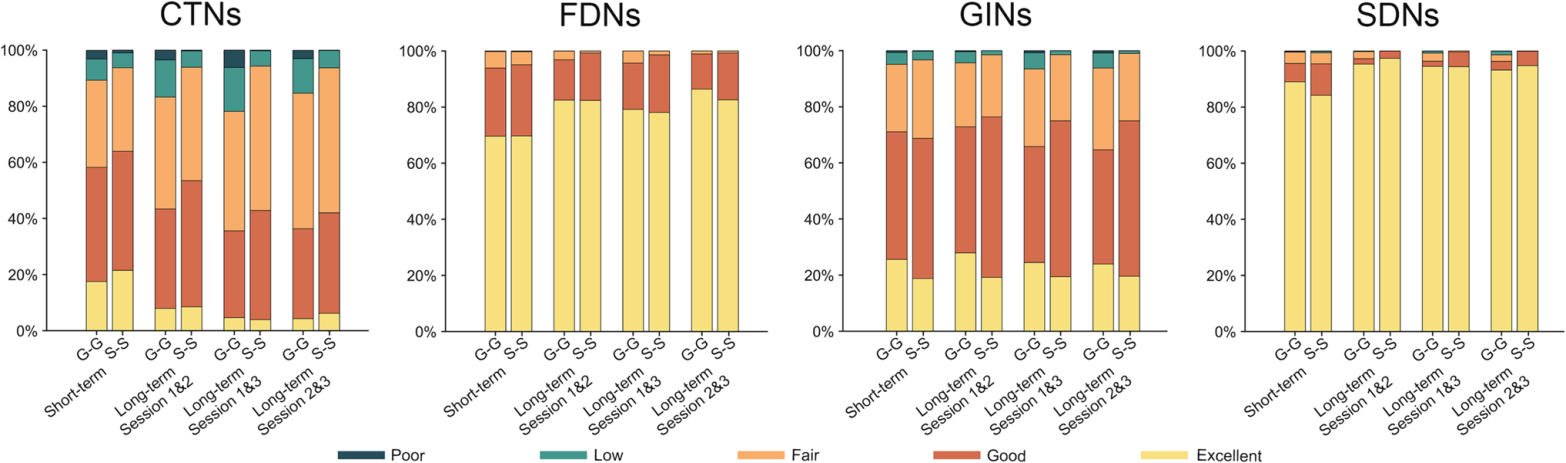
Proportions of edges showing different levels of TRT reliability. A considerable proportion of edges in the gyral and sulcal morphological brain networks exhibited good to excellent TRT reliability for the FDNs, GINs, and SDNs regardless of the time interval of scan-rescan. However, the proportions were obviously lower for the CTNs. CTNs, cortical thickness-based networks; FDNs, fractal dimension-based networks; GINs, gyrification index-based networks; SDNs, sulcal depth-based networks; G-G, gyri-gyri; S-S, sulci-sulci.

**Fig. 5:**
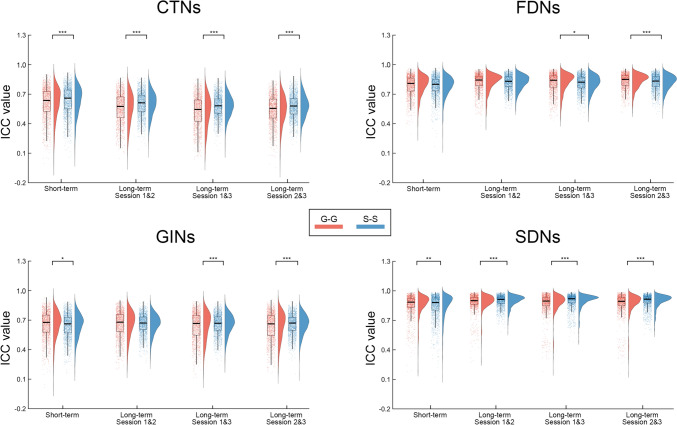
Differences in the TRT reliability between gyral and sulcal morphological brain networks. Compared to the sulcal networks, the gyral networks showed lower short-term TRT reliability for the CTNs, but higher short-term TRT reliability for the GINs and SDNs. With regard to long-term TRT reliability, the gyral networks were significantly lower for the CTNs, GINs, and SDNs, but higher for the FDNs than the sulcal networks. CTNs, cortical thickness-based networks; FDNs, fractal dimension-based networks; GINs, gyrification index-based networks; SDNs, sulcal depth-based networks; G-G, gyri-gyri; S-S, sulci-sulci; ICC, intra-class correlation. *P < 0.05; **P < 0.01; ***P < 0.001 (permutation test; 1,653 G-G edges *vs* 1,891 S-S edges).

Fig. S3 shows both the short-term and long-term TRT reliability of each edge in the G-G and S-S morphological brain networks. A considerable proportion of edges exhibited good to excellent reliability regardless of the time interval of scan-rescan and type of morphological brain networks (Fig. 4). The proportions were particularly high for the FDNs and SDNs. For example, 95.5% of edges in the G-G networks and 95.4% of edges in the S-S networks had a short-term ICC larger than 0.6 for the SDNs. Nonetheless, statistical comparisons revealed that both the short-term and long-term TRT reliability differed significantly between the G-G and S-S morphological brain networks. Specifically, for the short-term TRT reliability, edges in the G-G networks were more reliable for the GINs (*P* = 0.011) and SDNs (*P* = 0.002), but less reliable for the CTNs (*P* <0.001) than those in the S-S networks (Fig. 5). With regard to the long-term TRT reliability, edges in the G-G networks were more reliable for the FDNs (*P*_sessions 1&3_ = 0.026 and *P*_sessions 2&3_ = 0), but less reliable for the CTNs (*P* <0.001 for each pair of sessions), GINs (*P*_sessions 1&3_ <0.001 and *P*_sessions 2&3_ <0.001), and SDNs (*P* <0.001 for each pair of sessions) than those in the S-S networks (Fig. 5).

### Different Associations with Behavior and Cognition Between Gyral and Sulcal Morphological Brain Networks

**Fig. 6:**
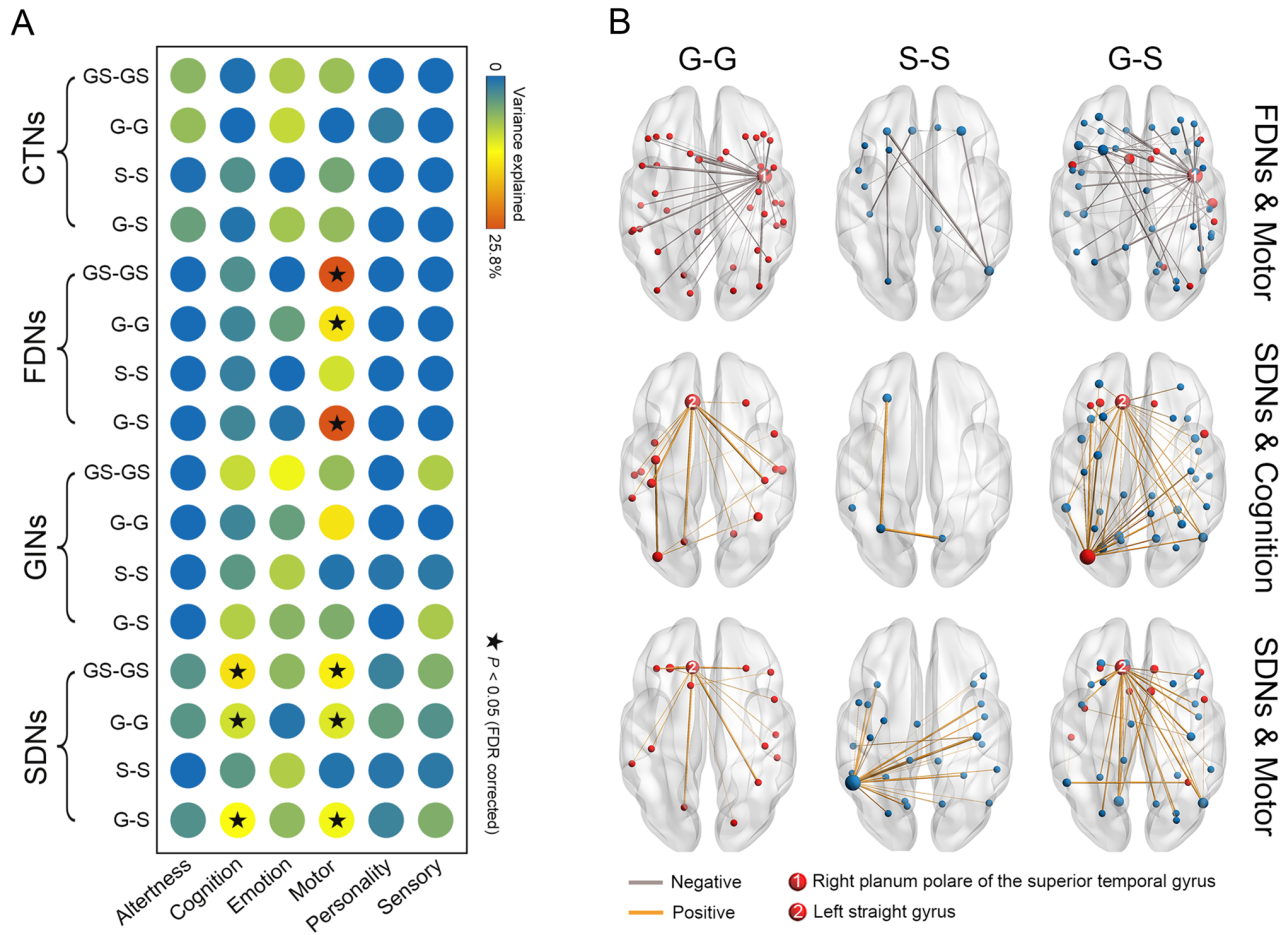
Behavioral and cognitive relevance of morphological brain networks. **A** The proportions of inter-individual variance explained in each behavioral and cognitive domain by different types of morphological networks and their subcomponents. The GS-GS, G-G, and G-S networks explained significant proportions of inter-individual variance in the Motor domain for the FDNs and in the Cognition and Motor domains for the SDNs. **B** Edges showing significant correlations with individual scores in the Motor and Cognition domains. Most of these edges were in the G-S networks, and were mainly linked to the right planum polare of the superior temporal gyrus or the left straight gyrus. Dot sizes are proportional to the number of edges linked to the nodes. Line widths are proportional to the correlation coefficients. CTNs, cortical thickness-based networks; FDNs, fractal dimension-based networks; GINs, gyrification index-based networks; SDNs, sulcal depth-based networks; GS-GS, gyri&sulci-gyri&sulci; G-G, gyri-gyri; S-S, sulci-sulci; G-S, gyri-sulci.

Fig. 6A presents the extent to which inter-individual variance in behavior and cognition is explained by the GS-GS morphological brain networks and their subcomponents. The GS-GS morphological brain networks explained significant proportions of inter-individual variance in the Motor domain for the FDNs (*P* = 0.006) and in the Cognition (*P* = 0.009) and Motor (*P* = 0.009) domains for the SDNs. The subcomponent analysis revealed similar results for the G-G and G-S networks (*P* <0.05, FDR corrected). Notably, the proportions of inter-individual variance explained by the G-S networks were obviously higher than those explained by the G-G networks.

We further identified edges that exhibited significant correlations with the Motor domain for the FDNs (95 edges; *P* <0.05, TFNBS corrected) and with the Cognition (67 edges; *P* <0.05, TFNBS corrected) and Motor (67 edges; *P* <0.05, TFNBS corrected) domains for the SDNs (Fig. 6B). For each of the three sets of edges, most were in the G-S networks (46, 48.4%; 47, 70.1%; 31, 46.3%), followed by in the G-G (the first set: 39, 41.1%; the second set: 17, 25.4%) or S-S (the third set: 23, 34.3%) networks. Interestingly, the edges were mainly associated with the right planum polare of the superior temporal gyrus for the FDNs (38 G-G edges + 28 G-S edges = 66 edges, 69.5%) and the left straight gyrus for the SDNs (Cognition: 13 G-G edges + 20 G-S edges = 33 edges, 49.3%; Motor: 11 G-G edges + 20 G-S edges = 31 edges, 46.3%).

### Different Susceptibilities to MDD Between Gyral and Sulcal Morphological Brain Networks

**Fig. 7:**
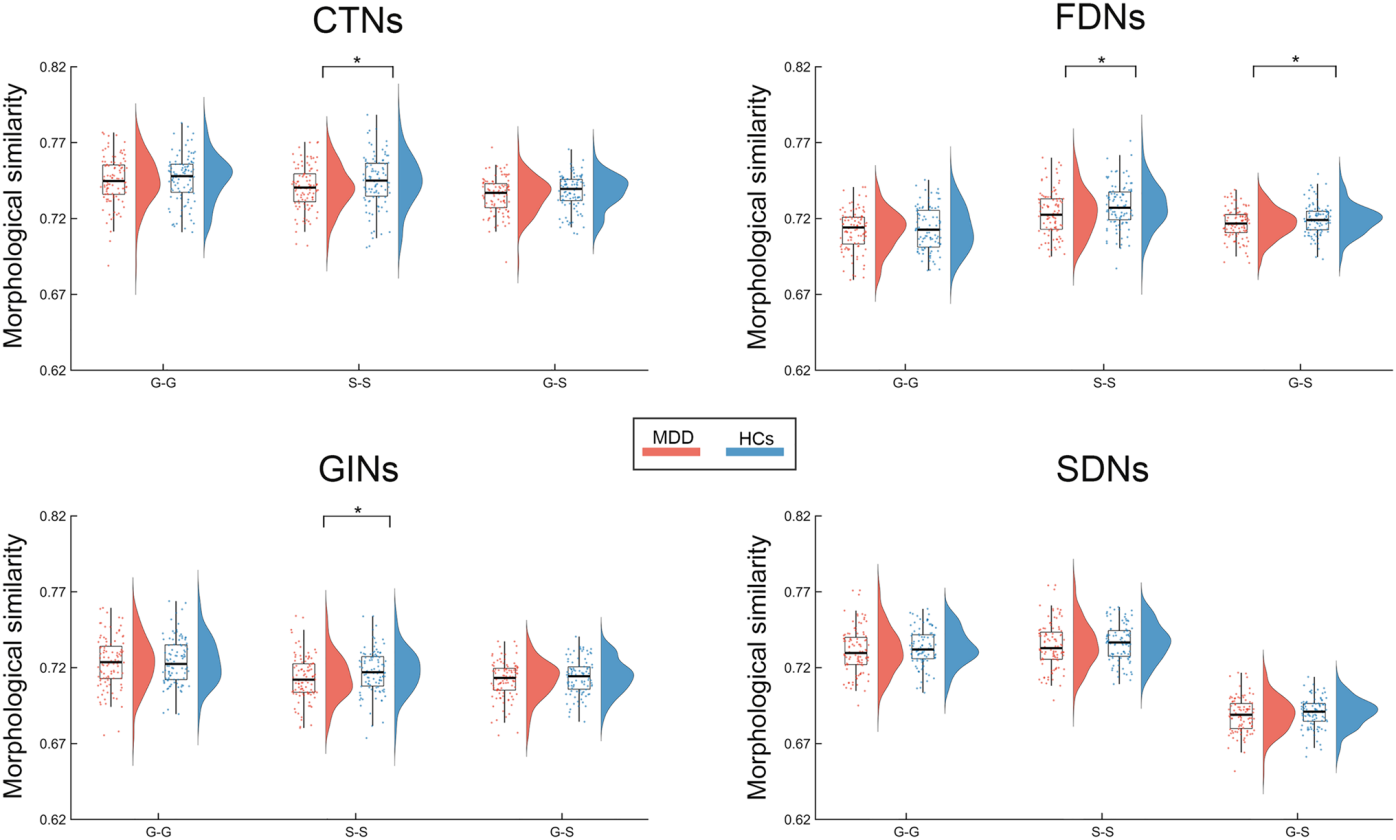
Differences in the mean morphological similarity in gyral and sulcal morphological brain networks between the MDD patients and controls. Compared to the controls, the MDD patients exhibited a significant decrease in the mean morphological similarity in the S-S networks for the CTNs, FDNs, and GINs, and in the G-S networks for the FDNs. CTNs, cortical thickness-based networks; FDNs, fractal dimension-based networks; GINs, gyrification index-based networks; SDNs, sulcal depth-based networks; G-G, gyri-gyri; S-S, sulci-sulci; G-S, gyri-sulci; MDD, major depressive disorder; HCs, healthy controls. *P <0.05 (permutation test; 100 patients *vs* 99 controls).

For the mean morphological similarity in the GS-GS networks, only the FDNs exhibited a significant decrease in MDD patients in comparison to the controls (*P* = 0.034) (Fig. S4). Further between-group comparisons of the mean morphological similarity in each subcomponent of the GS-GS morphological brain networks revealed that the MDD patients had a significant decrease in the S-S networks for the CTNs (*P* = 0.033), FDNs (*P* = 0.023), and GINs (*P* = 0.027), and in the G-S networks for the FDNs (*P* = 0.029) (Fig. 7). No significant correlations were found for these alterations with the HAMD scores or course of disease of the patients (*P* >0.05).

## Discussion

In this study, we systematically investigated the gyral and sulcal effects on morphological brain networks. We found that (1) compared to the S-S networks, the G-G networks showed higher morphological similarity for the CTNs and GINs, but lower morphological similarity for the FDNs; (2) although both the G-G and S-S networks exhibited the small-world organization, the former were less segregated for the CTNs, GINs, and SDNs, more integrated for the CTNs and GINs, and less integrated for the FDNs than the latter; (3) relative to the S-S networks, the G-G networks showed lower short-term TRT reliability for the CTNs and long-term TRT reliability for the CTNs, GINs, and SDNs, but higher short-term TRT reliability for the GINs and SDNs and long-term TRT reliability for the FDNs; (4) the G-G networks and connections between gyral and sulcal regions significantly explained inter-individual variance in the Cognition and Motor domains for the FDNs and SDNs; and (5) only the S-S networks exhibited morphological similarity reductions in the MDD patients for the CTNs, FDNs, and GINs. These findings deepen our understanding of the morphological architecture of the human brain from the perspective of large-scale networks.

### Different Levels of Morphological Similarity Between Gyral and Sulcal Morphological Brain Networks

Compared to the S-S networks, the G-G networks exhibited significantly higher morphological similarity for the CTNs and GINs, suggesting more homogeneous morphology between gyral than sulcal regions. A previous diffusion tensor imaging study reported that axonal fibers connected to gyri were significantly denser than those connected to sulci [14]. Evidence from functional MRI studies showed that gyri-gyri pairs had stronger functional connectivity than sulci-sulci pairs [11–13]. Our results are consistent with these previous findings, which collectively suggest a higher degree of information exchange and integration among gyral than sulcal regions of the brain. Interestingly, we found that both the G-G and S-S networks had significantly higher morphological similarity than connections linking gyri and sulci for the CTNs, GINs, and SDNs. These findings are not surprising given the substantial differences in cytoarchitecture between gyral and sulcal regions. For instance, the somata and arbors of pyramidal neurons are extended in gyri but compressed in sulci [51]. It should be noted that the FDNs exhibited a different pattern characterized by significantly higher morphological similarity between sulci but lower morphological similarity between gyri in comparison with connections between gyral and sulcal regions. Given that FD reflects the spatial complexity of cortical folding, these findings may imply that cortical folding complexity varies the most between gyral regions, which may be (partially) due to the extended somata and arbors of pyramidal neurons in gyral regions.

### Different Small-world Parameters Between Gyral and Sulcal Morphological Brain Networks

We found that both the G-G and S-S networks exhibited the small-world organization regardless of the morphological feature based on which they were constructed. This is consistent with a previous diffusion tensor imaging study, which also observed the small-world organization for both the G-G and S-S networks [13]. The small-world organization is a universal organizational principle of human brain networks [2, 52], which confers the human brain with the capacity for both specialized and integrated information processing. However, compared to the S-S networks, the G-G networks had significantly lower clustering coefficients for the CTNs, GINs, and SDNs and lower characteristic path lengths for the CTNs and GINs. The clustering coefficient quantifies the extent of local interconnectivity and is a measure of functional segregation, while characteristic path length reflects the capability of overall routing efficiency and is an index of functional integration. Thus, our findings suggest a less segregated but more integrated architecture of the G-G than S-S networks. These findings are reasonable given the observed significantly higher morphological similarity within the G-G than S-S networks for the CTNs and GINs. Previous evidence from functional brain network studies has shown that the G-G networks have higher economic properties and small-worldness than the S-S networks [13], and gyri act as global functional centers whereas sulci primarily function as local information exchange units [10, 11, 13, 53]. Here, our findings provide anatomical support for the crucial roles of gyri in global information integration from the perspective of inter-regional coordination of brain morphology.

### Different Degrees of TRT Reliability Between Gyral and Sulcal Morphological Brain Networks

We found that both the G-G and S-S networks exhibited high TRT reliability regardless of the time interval of scan-rescan and the morphological feature based on which they were constructed. These findings are consistent with our previous studies of whole-brain morphological brain networks [19, 35]. Nevertheless, significant differences were found between the G-G and S-S networks. Specifically, compared with the G-G networks, the S-S networks showed significantly higher long-term TRT reliability for the CTNs, GINs, and SDNs but lower long-term TRT reliability for the FDNs. Previous studies have shown that sulcal morphological descriptors, such as length, depth, width, and surface, are significantly heritable across the cortex [54–56]. Thus, sulcal morphological features and their derived networks may undergo fewer plastic changes in response to environmental variation and skill learning. In addition, a recent study found that during the normal aging process, gyral and sulcal changes in CT and intrinsic curvature exhibited different patterns [57]. The differences in heritability and aging patterns between gyri and sulci may contribute to some extent to the observed differences in the long-term TRT reliability between the gyral and sulcal networks. Long-term TRT reliability is important for methods and measures when using them to identify biomarkers for brain disorders. However, longer data acquisition intervals have been found to reduce the TRT reliability of morphological brain networks [35]. Based on our results, we suggest that for most morphological features the S-S networks may serve as a good candidate for studies aiming at identifying stable biomarkers for brain disorders.

### Different Capabilities of Gyral and Sulcal Morphological Brain Networks in Explaining Behavioral and Cognitive Variance

Consistent with our previous study [25], we found that the morphological brain networks explained significant proportions of inter-individual variance in the Cognition and Motor domains. Going deeper on this point, our subcomponent analysis revealed that it was the connections in the G-G networks and connections between the gyral and sulcal regions that contributed to the associations between the morphological brain networks and behavior and cognition. This is consistent with a previous functional MRI study, which found that G-S functional connectivity was able to predict various cognitive behaviors [58]. Further edgewise correlation analysis revealed that connections showing significant correlations with individual scores of the Cognition and Motor domains were mainly linked to gyral regions. All these findings point toward the important role of gyral regions in supporting cognition and motor functions. These findings are in line with previous task functional MRI studies, which have shown that (1) regions activated during various task conditions are located significantly more on gyral than sulcal regions [59]; (2) different functional networks spatially converge significantly more on gyral than sulcal regions [60]; and (3) gyral regions have smaller signal representation residual than sulcal regions, indicative of more involvement of gyral regions in global functions of the brain and interregional communications [10]. Interestingly, we noted that most connections that were significantly correlated to the Motor domain for the FDNs were linked to the planum polare of the superior temporal gyrus, and most connections that were significantly correlated to the Cognition and Motor domains for the SDNs were linked to the straight gyrus. Previous studies have shown that the superior temporal gyrus is vital for processing motion produced by human agents [61, 62]. For the straight gyrus, several studies have found that its size and morphology (e.g., gray matter volume and surface area) are related to individual differences in memory and social function [63–65]. Moreover, the partial resection of the straight gyrus is shown to result in selective impairment of memory [66]. Expanding on these previous findings, our results provide new evidence for the cognitive relevance of these two regions from the network perspective of morphological similarity.

### Different Susceptibilities of Gyral and Sulcal Morphological Brain Networks to MDD

We found that only the S-S networks exhibited significantly decreased morphological similarity in the MDD patients for the CTNs, FDNs, and GINs. These findings suggest that the S-S networks are more susceptible to MDD. Previous evidence from studies of animals, computational models, and human postmortem data has shown that sulcal regions, compared with gyral areas, have greater physical strain in various kinds of injuries [67–69]. Moreover, sulcal regions are found to be more vulnerable to pathological tau protein deposition and related brain atrophy in traumatic brain injury and chronic traumatic encephalopathy [70–72]. These previous findings together with our results jointly suggest that the sulcal regions and their derived networks may be the preferential sites or measures to manifest alterations in neuropsychiatric disorders. Specifically, our observed morphological similarity reductions imply increased variation of sulcal morphology in MDD. According to the tension-based theory of morphogenesis [73], the increased variation might be related to deficits of white matter tracts linking sulcal regions in MDD. This speculation sounds plausible given recent findings that inter-regional morphological similarity is positively correlated to axonal tract-tracing connectivity [74, 75]. Nevertheless, further studies are required to directly examine the relationship between morphology-based network alterations and white-matter deficits in the context of MDD. In addition, given that there are a growing number of studies reporting alterations of sulcal morphology in various brain diseases, such as autism [76, 77], bipolar disorder [78], schizophrenia [79], and Alzheimer's disease [80], it is interesting for future studies to explore whether sulcal morphological brain networks are altered in these diseases and to determine the extent to which the alterations resemble each other.

### Morphological Feature-dependent Differences Between Gyral and Sulcal Morphological Brain Networks

Although significant differences were found between the G-G and S-S networks in all aspects examined in this study, the patterns of the differences varied between the cortical morphological networks constructed with different morphological features (i.e., CT, FD, GI, and SD). Presumably, the major reason may be the different cellular mechanisms among these morphological features. For example, CT is thought to reflect the arrangement, size, and density of neurons, neuroglia, and nerve fibers [81], while FD reflects the spatial complexity of cortical folding by integrating folding frequency, SD, gyral shape, and CT [82]. Moreover, these morphological features demonstrate different sensitivities to detect age-related changes [83] and regional heterogeneity in age-related changes varies across them [32]. All the above-mentioned differences may contribute to, at least to some extent, previously reported differences among the cortical morphological brain networks constructed with the four morphological features [19, 25, 35, 36] and the different patterns of network differences between gyri and sulci as observed in this study.

### Limitations and Future Directions

This study had several limitations. First, morphological brain networks were constructed using 4 morphological features computationally available in the CAT12 toolbox. Beyond these morphological features, future studies are required to explore the gyral and sulcal effects on morphological brain networks built based on other morphological features, such as surface area and mean curvature. Second, inter-regional morphological similarity was estimated based on the divergence-based method. In addition to this method, morphological similarity can be estimated through correlation-based [84] or distance-based [85] methods. To more comprehensively unveil the gyral and sulcal effects, future studies are warranted to construct morphological brain networks using other methods. Third, we found that morphological similarity in the S-S morphological brain networks was more susceptible than that in the G-G networks to MDD. It is interesting to investigate whether other psychiatric disorders also selectively affect gyral or sulcal morphological brain networks. Finally, for a better understanding of the network differences between gyri and sulci, it is important to conduct multimodal studies by jointly analyzing brain structure, morphology, and function from an integration perspective.

## Conclusion

In summary, this study examined the gyral and sulcal effects on different types of single-subject morphological brain networks. Significant differences were found between the gyral and sulcal networks in multiple aspects, including the level of morphological similarity, small-world organization, TRT reliability, association with behavior and cognition, and susceptibility to MDD. These findings deepen our understanding of the influence of cortical folding patterns on the network organization of the human brain.

## Supplementary Information

Below is the link to the electronic supplementary material.

Supplementary file1 (PDF 670 KB)

## Data Availability

All data that support the findings of this study are from publicly available datasets.
