# Supplementary material for: Cortical Morphological Networks Differ Between Gyri and Sulci
Source: Neurosci Bull. 2024 Jul 23;41(1):46–60. doi: 10.1007/s12264-024-01262-7 (PMC11748734; doi:10.1007/s12264-024-01262-7)
Supplement: Supplementary file 1 — Supplementary file1 (PDF 670 KB) [file 12264_2024_1262_MOESM1_ESM.pdf]

Supplementary Materials

Supplementary Tables

Table S1 Behavioral and cognitive information included in this study for the HCP dataset (unrelated participants)

Domain	Subdomain (Test)	Item	Score
Alertness	Cognitive Status (Mini Mental Status Examination)	MMSE_Score	29.02 ± 1.09 (23–30)
	Sleep (Pittsburgh Sleep Quality Index)	PSQI_Score	4.93 ± 2.84 (0–15)
Cognition	Episodic Memory (Picture Sequence Memory)	PicSeq_Unadj	111.44 ± 13.62 (76.42–135.55)
	Cognitive Flexibility (Dimensional Change Card Sort)	CardSort_Unadj	114.66 ± 10.47 (85.01–143.94)
	Inhibition (Flanker Inhibitory Control and Attention Task)	Flanker_Unadj	111.08 ± 10.05 (84.90–142.11)
	Fluid Intelligence (Penn Progressive Matrices)	PMAT24_A_CR	16.83 ± 4.90 (4–24)
	Language/Reading Decoding (Oral Reading Recognition)	ReadEng_Unadj	116.34 ± 10.35 (86.20–150.71)
	Language/Vocabulary Comprehension (Picture Vocabulary)	PicVocab_Unadj	116.4 ± 9.42 (92.39–153.09)
	Processing Speed (Pattern Comparison Processing Speed)	ProcSpeed_Unadj	114.61 ± 15.58 (6.09–154.69)
	Self-regulation/Impulsivity (Delay Discounting)	DDisc_AUC_200	0.25 ± 0.19 (0.02–0.98)
		DDisc_AUC_40K	0.50 ± 0.28 (0.02–0.98)
	Spatial Orientation (Variable Short Penn Line Orientation Test)	VSPLOT_TC	14.81 ± 4.55 (2–26)
		VSPLOT_OFF	24.54 ± 15.64 (5–134)
		VSPLOT_CRTE	1164.85 ± 365.74 (493.07–2812.22)
	Sustained Attention (Short Penn Continuous Performance Test)	SCPT_SEN	0.95 ± 0.08 (0–1)
		SCPT_SPEC	0.95 ± 0.04 (0.76–1)
	Verbal Episodic Memory (Penn Word Memory Test)	IWRD_TOT	35.74 ± 2.89 (22–40)
	Working Memory (List Sorting)	ListSort_Unadj	111.31 ± 11.08 (80.79–144.50)
	Cognition Summary Scores (Cognitive Function Composite Scores)	CogFluidComp_Unadj	114.71 ± 11.93 (84.48–145.08)
		CogEarlyComp_Unadj	116.54 ± 11.10 (85.63–153.55)
		CogTotalComp_Unadj	121.13 ± 14.73 (84.55–153.36)
		CogCrystalComp_Unadj	117.26 ± 9.60 (90.95–147.56)
Emotion	Emotion Recognition (Penn Emotion Recognition Test)	ER40_CR	35.65 ± 2.55 (24–40)
		ER40ANG	6.79 ± 1.07 (2–8)
		ER40FEAR	7 ± 1.13 (0–8)
		ER40NOE	7.15 ± 1.21 (0–8)
		ER40SAD	6.75 ± 1.20 (1–8)
	Negative Affect (Self-report Questionnaire)	AngAffect_Unadj	48.19 ± 8.64 (28.60–85.40)
		AngHostil_Unadj	50.86 ± 8.77 (36.60–74)
		AngAggr_Unadj	51.94 ± 8.82 (43.4–83.10)
		FearAffect_Unadj	50.31 ± 8.17 (32.90–84.90)
		FearSomat_Unadj	51.73 ± 8.38 (40.10–87.50)
		Sadness_Unadj	46.77 ± 8.20 (34.20–76.90)
	Psychological Well-being (Self-report uestionnaire)	LifeSatisf_Unadj	54.48 ± 9.56 (24.20–74.60)
		MeanPurp_Unadj	51.26 ± 8.69 (30.90–71.60)
		PosAffect_Unadj	50.44 ± 7.58 (25.40–71.60)

	Social Relationships (Self-report Questionnaire)	Friendship_Unadj	50.54 ± 9.39 (20.80–66.50)
		Loneliness_Unadj	51.35 ± 8.69 (37.60–77.40)
		PercHostil_Unadj	48.40 ± 8.84 (33.50–80.20)
		PercReject_Unadj	48.26 ± 8.54 (35.90–72.60)
		EmotSupp_Unadj	51.20 ± 9.66 (15.90–62.50)
		InstruSupp_Unadj	47.67 ± 9.47 (22.10–62.90)
	Stress and Self-Efficacy (Self-report Questionnaire)	PercStress_Unadj	48.45 ± 9.82 (22.40–80.50)
		SelfEff_Unadj	50.84 ± 8.29 (31.30–68.40)
Motor	Endurance (2-minute Walk Test)	Endurance_Unadj	109.65 ± 12.02 (81.34–145.10)
	Locomotion (4-meter Walk Test)	GaitSpeed_Comp	1.32 ± 0.19 (0.79–2)
	Dexterity (9-hole Pegboard)	Dexterity_Unadj	112.03 ± 10.92 (85.15–148.67)
	Strength (Grip Strength Dynamometry)	Strength_Unadj	117.00 ± 11.16 (88.80–154.59)
Personality	Five Factor Model (NEO-FFI)	NEOFAC_A	33.11 ± 5.67 (10–47)
		NEOFAC_O	28.62 ± 5.83 (14–45)
		NEOFAC_C	34.64 ± 5.88 (16–48)
		NEOFAC_N	17.13 ± 7.79 (0–43)
		NEOFAC_E	30.60 ± 6.02 (10–47)
Sensory	Audition (Words in Noise)	Noise_Comp	4.43 ± 1.51 (-0.4–11.6)
	Olfaction (Odor Identification Test)	Odor_Unadj	109.72 ± 8.94 (93.38–122.25)
	Pain (Pain Intensity and Interference Surveys)	PainIntens_RawScore	1.45 ± 1.87 (0–10)
		PainInterf_Tscore	45.93 ± 7.89 (38.60–71.60)
	Taste (Regional Taste Intensity Test)	Taste_Unadj	95.06 ± 14.44 (67.48–134.65)
	Contrast Sensitivity (Mars Contrast Sensitivity)	Mars_Log_Score	1.85 ± 0.63 (1.56–15)
		Mars_Final	1.83 ± 0.63 (1.08–14.96)

Data are presented as the mean ± SD (minimum–maximum).

Table S2 The gyral and sulcal regions included in this study

Class	Abbreviation	Full name
Gyrus	Cingul-Post-dorsal	Posterior-dorsal part of the cingulate gyrus (dPCC)
Gyrus	Cingul-Post-ventral	Posterior-ventral part of the cingulate gyrus (vPCC, isthmus of the cingulate gyrus)
Gyrus	Cuneus	Cuneus
Gyrus	Front_inf-Opercular	Opercular part of the inferior frontal gyrus
Gyrus	Front_inf-Orbital	Orbital part of the inferior frontal gyrus
Gyrus	Front_inf-Triangul	Triangular part of the inferior frontal gyrus
Gyrus	Front_middle	Middle frontal gyrus (F2)
Gyrus	Front_sup	Superior frontal gyrus (F1)
Gyrus	Insular_short	Short insular gyri
Gyrus	Occipital_middle	Middle occipital gyrus (O2, lateral occipital gyrus)
Gyrus	Occipital_sup	Superior occipital gyrus (O1)
Gyrus	Oc-temp_lat-fusifor	Lateral occipito-temporal gyrus (fusiform gyrus, O4-T4)
Gyrus	Oc-temp_med-Lingual	Lingual gyrus, ligual part of the medial occipito-temporal gyrus, (O5)
Gyrus	Oc-temp_med-Parahip	Parahippocampal gyrus, parahippocampal part of the medial occipito-temporal gyrus, (T5)
Gyrus	Orbital	Orbital gyri
Gyrus	Pariet_inf-Angular	Angular gyrus
Gyrus	Pariet_inf-Supramar	Supramarginal gyrus
Gyrus	Parietal_sup	Superior parietal lobule (lateral part of P1)
Gyrus	Postcentral	Postcentral gyrus
Gyrus	Precentral	Precentral gyrus
Gyrus	Precuneus	Precuneus (medial part of P1)
Gyrus	Rectus	Straight gyrus, Gyrus rectus
Gyrus	Subcallosal	Subcallosal area, subcallosal gyrus
Gyrus	Temp_sup-G_T_transv	Anterior transverse temporal gyrus (of Heschl)
Gyrus	Temp_sup-Lateral	Lateral aspect of the superior temporal gyrus
Gyrus	Temp_sup-Plan_polar	Planum polare of the superior temporal gyrus
Gyrus	Temp_sup-Plan_tempo	Planum temporale or temporal plane of the superior temporal gyrus
Gyrus	Temporal_inf	Inferior temporal gyrus (T3)

Gyrus	Temporal_middle	Middle temporal gyrus (T2)
Sulcus	Calcarine	Calcarine sulcus
Sulcus	Central	Central sulcus (Rolando’s fissure)
Sulcus	Cingul-Marginalis	Marginal branch (or part) of the cingulate sulcus
Sulcus	Circular_insula_ant	Anterior segment of the circular sulcus of the insula
Sulcus	Circular_insula_inf	Inferior segment of the circular sulcus of the insula
Sulcus	Circular_insula_sup	Superior segment of the circular sulcus of the insula
Sulcus	Collat_transv_ant	Anterior transverse collateral sulcus
Sulcus	Collat_transv_post	Posterior transverse collateral sulcus
Sulcus	Front_inf	Inferior frontal sulcus
Sulcus	Front_middle	Middle frontal sulcus
Sulcus	Front_sup	Superior frontal sulcus
Sulcus	Interm_prim-Jensen	Sulcus intermedius primus (of Jensen)
Sulcus	Intrapariet_and_P_trans	Intraparietal sulcus (interparietal sulcus) and transverse parietal sulci
Sulcus	Oc_middle_and_Lunatus	Middle occipital sulcus and lunatus sulcus
Sulcus	Oc_sup_and_transversal	Superior occipital sulcus and transverse occipital sulcus
Sulcus	Occipital_ant	Anterior occipital sulcus and preoccipital notch (temporo-occipital incisure)
Sulcus	Oc-temp_lat	Lateral occipito-temporal sulcus
Sulcus	Oc-temp_med_and_Lingual	Medial occipito-temporal sulcus (collateral sulcus) and lingual sulcus
Sulcus	Orbital_lateral	Lateral orbital sulcus
Sulcus	Orbital_med-olfact	Medial orbital sulcus (olfactory sulcus)
Sulcus	Orbital-H_Shaped	Orbital sulci (H-shaped sulci)
Sulcus	Parieto_occipital	Parieto-occipital sulcus (or fissure)
Sulcus	Pericallosal	Pericallosal sulcus (S of corpus callosum)
Sulcus	Postcentral	Postcentral sulcus
Sulcus	Precentral-inf-part	Inferior part of the precentral sulcus
Sulcus	Precentral-sup-part	Superior part of the precentral sulcus
Sulcus	Suborbital	Suborbital sulcus (sulcus rostrales, supraorbital sulcus)
Sulcus	Subparietal	Subparietal sulcus
Sulcus	Temporal_inf	Inferior temporal sulcus

Sulcus	Temporal_sup	Superior temporal sulcus (parallel sulcus)
Sulcus	Temporal_transverse	Transverse temporal sulcus

Supplementary Figures

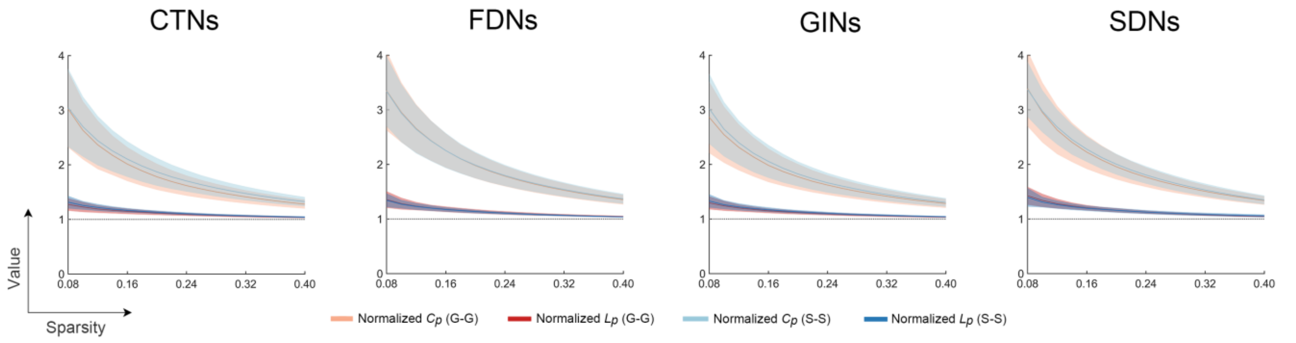

Fig. S1 The small-world organization of gyral and sulcal morphological brain networks. Both the gyral and sulcal morphological brain networks exhibited typical small-world organization (i.e., normalized clustering coefficient > 1 and normalized characteristic path length ~1) over the entire sparsity range regardless of the morphological features based on which the networks were constructed. CTNs, cortical thickness-based networks; FDNs, fractal dimension-based networks; GINs, gyrification index-based networks; SDNs, sulcal depth-based networks; G-G, gyri-gyri; S-S, sulci-sulci; C_p, clustering coefficient; L_p, characteristic path length.

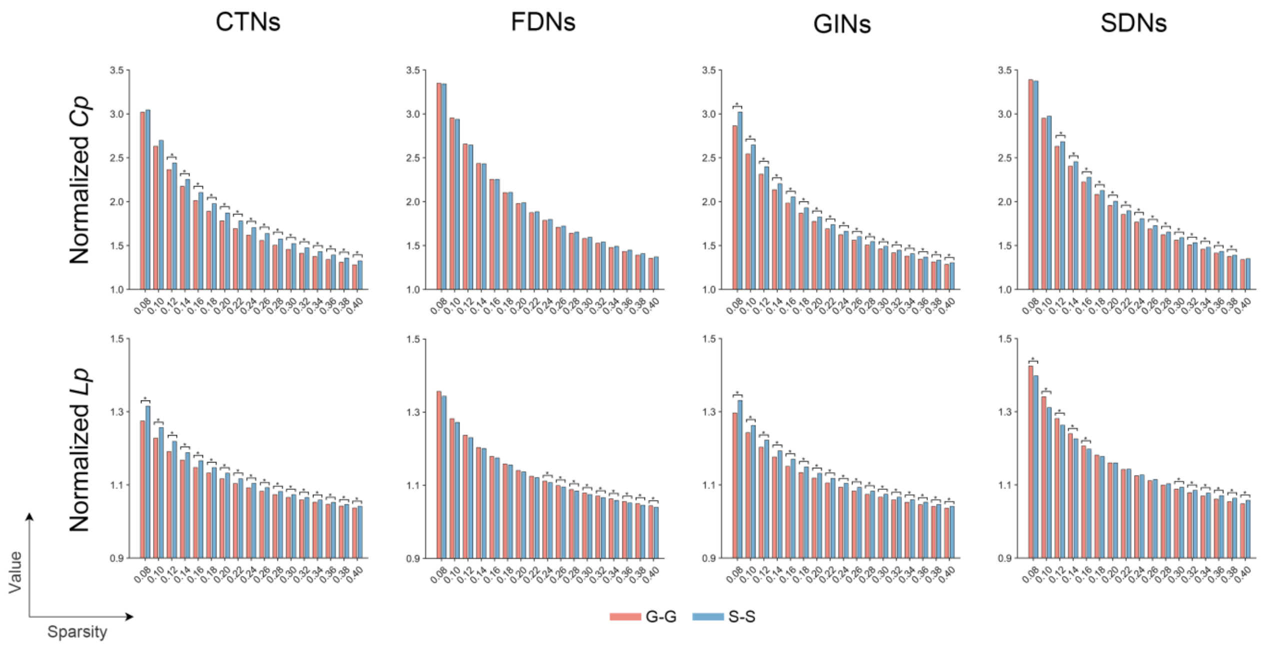

Fig. S2 Differences in the small-world parameters between gyral and sulcal morphological brain networks at each sparsity level. CTNs, cortical thickness-based networks; FDNs, fractal dimension-based networks; GINs, gyrification index-based networks; SDNs, sulcal depth-based networks; G-G, gyri-gyri; S-S, sulci-sulci; C_p, clustering coefficient; L_p, characteristic path length. *$P < 0.05$, FDR corrected across different sparsity levels (permutation test; 444 G-G networks *vs* 444 S-S networks).

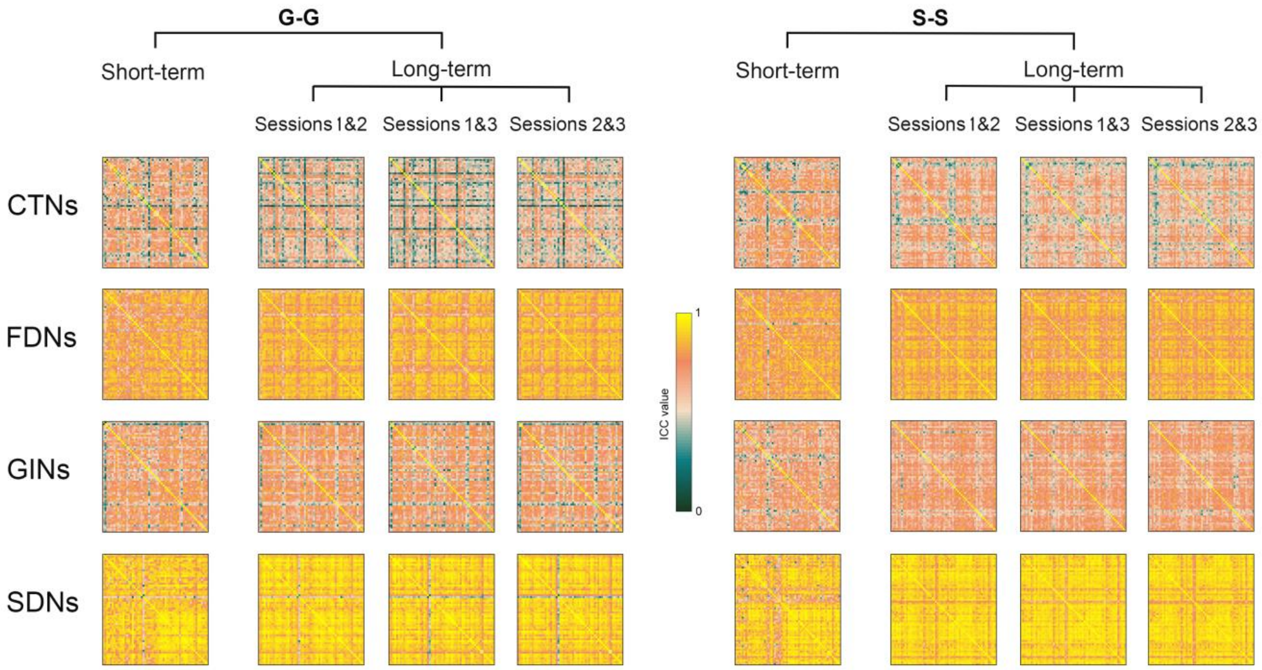

Fig. S3 The TRT reliability of gyral and sulcal morphological brain networks. High both short-term and long-term TRT reliability were observed for most edges in the gyral and sulcal morphological brain networks for the FDNs and SDNs. However, a small portion of edges in the gyral and sulcal morphological brain networks exhibited relatively low reliability for the CTNs and GINs regardless of the time interval of scan-rescan. CTNs, cortical thickness-based networks; FDNs, fractal dimension-based networks; GINs, gyrification index-based networks; SDNs, sulcal depth-based networks; G-G, gyri-gyri; S-S, sulci-sulci; ICC, intra-class correlation.

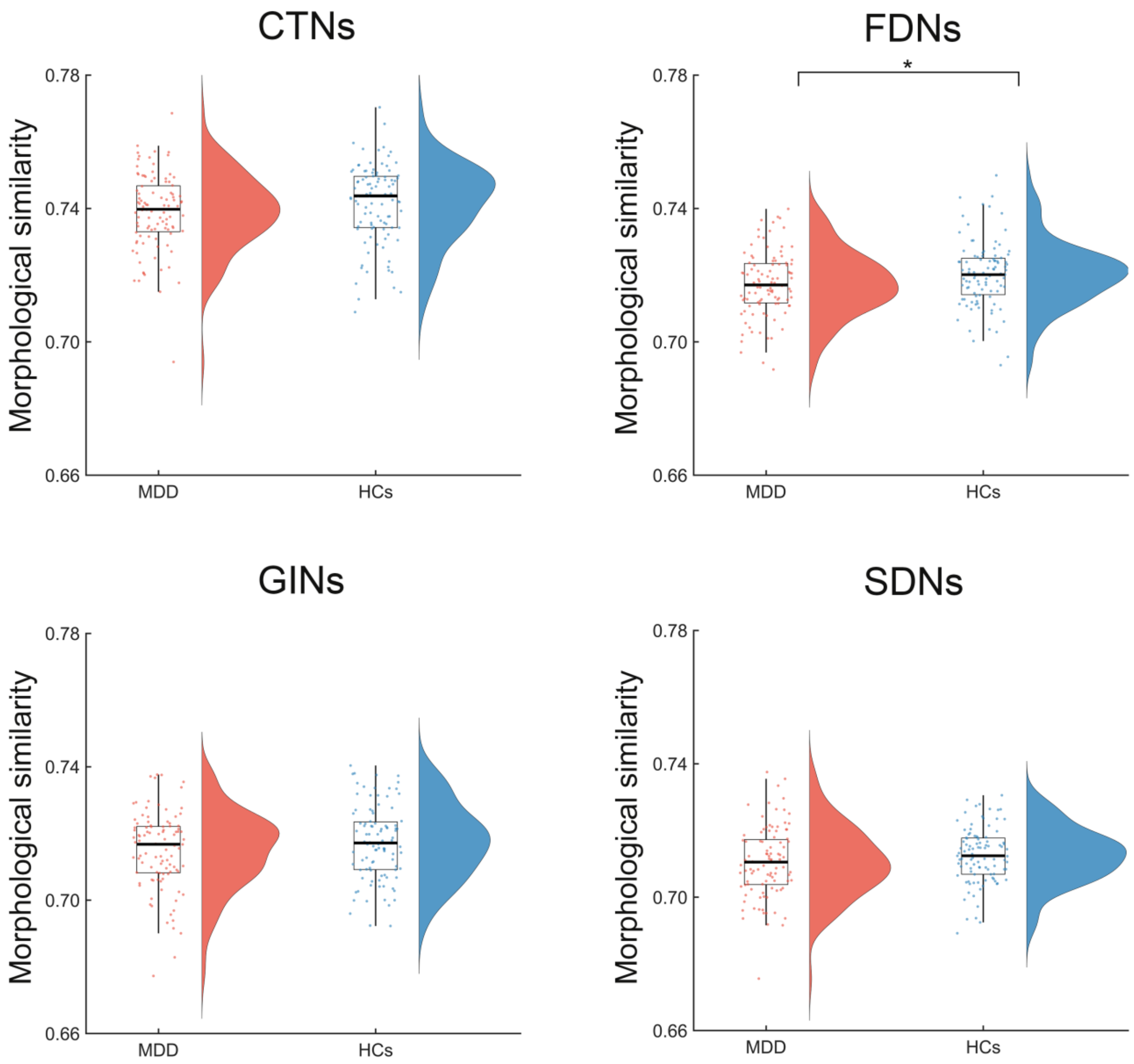

Fig. S4 Differences in the mean morphological similarity in whole-brain morphological brain networks between the MDD patients and HCs. Compared to the HCs, the MDD patients exhibited a significant decrease in the mean morphological similarity only for the FDNs. CTNs, cortical thickness-based networks; FDNs, fractal dimension-based networks; GINs, gyrification index-based networks; SDNs, sulcal depth-based networks; MDD, major depressive disorder; HCs, healthy controls. **P* < 0.05 (permutation test; 100 patietns *vs* 99 controls).
